# Maternal Manganese Restriction Increases Susceptibility to High-Fat Diet-Induced Dyslipidemia and Altered Adipose Function in WNIN Male Rat Offspring

**DOI:** 10.1155/2011/486316

**Published:** 2011-10-11

**Authors:** Manisha Ganeshan, Pothaganti B. Sainath, Inagadapa J. Naga Padmavathi, Lagishetty Venu, Yedla Durga Kishore, Kalle Anand Kumar, Nemani Harishanker, J. Srinivasa Rao, Manchala Raghunath

**Affiliations:** ^1^Division of Endocrinology and Metabolism, National Institute of Nutrition, Hyderabad 500 604, India; ^2^Department of Orthopaedic Surgery, David Geffen School of Medicine, University of California, Los Angeles, CA 90095, USA; ^3^National Centre for Laboratory Animal Sciences, National Institute of Nutrition, Hyderabad 500 604, India; ^4^Food Chemistry Division, National Institute of Nutrition, Hyderabad 500 604, India

## Abstract

Growth *in utero* is largely a reflection of nutrient and oxygen supply to the foetus. We studied the effects of Mn restriction *per se*, maternal Mn restriction, and postnatal high-fat feeding in modulating body composition, lipid metabolism and adipocyte function in Wistar/NIN (WNIN) rat offspring. Female weanling, WNIN rats received *ad libitum* for 4 months, a control or Mn-restricted diet and were mated with control males. Some restricted mothers were rehabilitated with control diet from conception (MnRC) or parturition (MnRP), and their offspring were raised on control diet. Some restricted offspring were weaned onto control diet (MnRW), while others continued on restricted diet throughout (MnR). A set of offspring from each group was fed high-fat diet from 9 months onwards. Body composition, adipocytes function, and lipid metabolism were monitored in male rat offspring at regular intervals. Maternal manganese restriction increased the susceptibility of the offspring to high-fat-induced adiposity, dyslipidaemia, and a proinflammatory state but did not affect their glycemic or insulin status.

## 1. Introduction

 Tissues and organs of the developing foetus go through critical periods of development [1] which may coincide with periods of rapid cell division. Exposure of the foetus to maternal malnutrition causes intrauterine growth retardation (IUGR) [2] leading to low birth weight whose prevalence varies from 13% to 30% in India. However, most animal models that studied the mechanistic basis of this relationship considered only the maternal deficiency of macronutrients. 

Micronutrients, especially minerals, play an important role in the structure and metabolic activities of animals and are important in their reproduction [3]. Despite this, the role of maternal micronutrient deficiencies in the etiology of adiposity and lipid metabolism in the offspring has not been studied well. We showed earlier that maternal mineral restriction induced irreversible alterations in body fat% and lipid metabolism in rat offspring, whereas maternal vitamin restriction induced similar but reversible changes [4, 5]. 

Manganese (Mn), an essential micronutrient for humans and animals, is an enzyme cofactor and a constituent of metalloenzymes [6]. It activates enzymes of fatty acid synthesis [7], hepatic gluconeogenesis [8], and is a critical component of manganese superoxide dismutase (MnSOD) involved in mitochondrial oxidant defense system. Although reports on Mn restriction *per se* are limited, many epidemiological studies have reported the importance of Mn as a supplement in reversing glucose intolerance induced by its deficiency [9]. However, the role of maternal manganese restriction on body composition and lipid metabolism in the offspring has not been deciphered yet. 

 Increase in metabolic disorders is attributed primarily to calorie-dense foods and decreased physical activity. Consumption of high-fat (HF) diet leads to increased energy intake, overweight, and obesity both in humans [10] and animals [11, 12]. Ready availability of HF foods is likely to contribute to the high prevalence of obesity in Western countries [13] and in developing countries, where traditional diets are being replaced by diets with HF content [14]. 

Excess of energy (more than required) is stored as fat, and thus long-term overconsumption of energy-rich foods leads to obesity. Several animal studies suggest that energy density, rather than simply an increased percentage of dietary fat, is the actual predisposing factor for weight gain [15]. Developmental programming may influence body composition through appetite regulation, epigenetic modification of key regulatory genes, altered fat deposition, and adipocyte metabolism [16]. Although body weight is tightly regulated, it has been shown that when animals or humans consume a diet with HF content on a regular basis, the amount of stored fat they maintain increases [17]. Studies in animals have shown that short-term HF feeding causes hepatic insulin resistance preceding the subsequent more long-term development of peripheral insulin resistance [18, 19]. 

In view of the foregone literature and unknown studies on the effect of maternal Mn restriction on body adiposity, the present study was conducted to validate/negate the hypothesis that maternal Mn restriction *per se* predisposes the WNIN rat offspring to altered body composition and increases its susceptibility to HF-induced adiposity and altered lipid metabolism in later life. 

## 2. Materials and Methods

The animal experiments were carried out in adherence to the “principles of laboratory animal care” (NIH publication no. 85-23, revised 1985) and with the approval of the ethical committee on animal experiments at National Institute of Nutrition, Hyderabad, India. 

Thirty, female, weanling WNIN rats obtained from the National Centre for Laboratory Animal Sciences, National Institute of Nutrition, Hyderabad, India, were divided into 2 groups of 6 and 24, housed individually in polypropylene cages with wire mesh bottom, maintained at 22 ± 2°C under standard lighting conditions (12 hr light/dark cycle) and had free access to deionised water. The group of 6 rats were fed *ad libitum*, a basal diet (based on the American Institute of Nutrition AIN-93G diet) [20] containing 8.92 mg of Mn/Kg diet, whereas the group of 24 rats were fed a Mn-restricted diet (same as basal diet but Mn salt was excluded from mineral mixture) with 0.33 mg of Mn/Kg diet (96% of Mn restriction compared to control diet). At the end of 4 months of feeding, blood was collected from the supraorbital sinus of rats fasted overnight to determine blood Mn levels and plasma lipid profile in WNIN female rats. 

**Figure 1:**
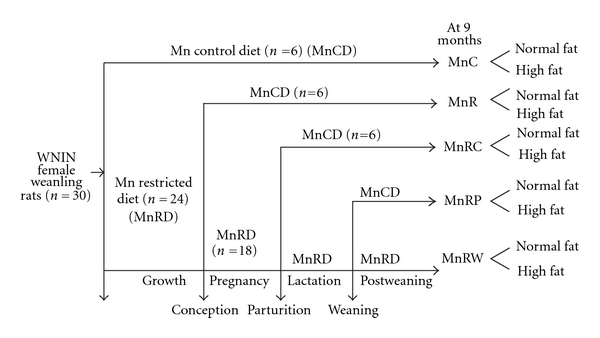
Schematic representation of the feeding protocol of different groups of WNIN rat mothers and their offspring. MnC: control group, MnR: manganese restricted group, MnRC: rehabilitation from conception group, MnRP: rehabilitation from parturition group, MnRW: rehabilitation from weaning group.

After assessing Mn status, rats were mated with control males (2 females to 1 male) and maintained on their respective diets during mating. After confirming conception, control rats received the control diet throughout their growth, gestation, and lactation, and their offspring were reared on control diet (MnC). On the other hand, one fourth (*n* = 6) each of the pregnant MnR dams were shifted to control diet from conception (MnRC) or parturition (MnRP), and their offspring weaned on to control diet. Remaining half of the pregnant MnR dams continued on restricted diet throughout lactation, and at weaning, half of their offspring were switched to control diet (MnRW), while the other half continued on restricted diet (MnR). A uniform litter size (8 offspring per mother—equal numbers of males and females wherever possible) was maintained in all groups from postnatal day 3. The feeding protocol is depicted schematically in Figure 1. At 9 months of age, the five groups of offspring, namely, MnC, MnR, MnRC, MnRP, and MnRW as described earlier, were further divided into another subgroup which had the same diet composition as the parent group but containing high fat in the diet. Body composition, adipose tissue function, and lipid metabolism were monitored in the offspring of both sexes at various time points; however, due to the similarity of the changes, the results pertaining only to the male offspring have been reported in this paper.

### 2.1. Preparation of High-Fat Diet

 To meet the criteria of a “high-fat diet”, 32%–35% of the energy in the diet should be derived from fats. In the preparation of the high-fat diet, energy from the carbohydrate component of the diet was reduced and added to that derived from fat as described earlier [21]. All other diet components were similar to that of the AIN-93G basal diet. The composition of the AIN 93G and the high-fat diet are given in Table 1. HF diet had 2 folds higher fat content compared to the AIN-93G diet. Comparison of energies obtained from the two different diets is given in Table 2.

### 2.2. Reproductive Performance

Percentage of animals conceived, weight gain during pregnancy, number of pups delivered, number of still births, and body weight of the offspring at birth/weaning were recorded to assess the effect(s) of chronic dietary Mn restriction *per se* on reproduction. 

### 2.3. Blood Manganese Levels

Manganese levels were determined, using graphite furnace atomic absorption spectrometer (GFS97 SOLAAR AA Series; Thermo Electron, Cheshire, Conn, USA) according to Mahalingam et al. [22] in the whole blood of the WNIN female rats (mothers just before mating) and in the offspring at quarterly intervals between 3 and 18 months of age. 

### 2.4. Body Composition

Body composition of the offspring was determined at the time points mentioned above, by total body electrical conductivity (TOBEC) measurement, and body fat% was computed as described by us earlier [5]. 

### 2.5. Adiposity Index

Adiposity index (AI), a measure of the total weight of the visceral fat depots (epididymal, retroperitoneal, and mesenteric) in the body, was determined according to Taylor and Phillips [23], using the formula: AI = (sum of the weights of the visceral fat depots/body weight) × 100.

### 2.6. Plasma and Tissue Adipocytokines

Adipocytokines were quantified in plasma and adipose tissue lysate [24] using the Milliplex MAP kits (7 plex kit) procured from M/S Millipore Corporation Ltd according to Allan et al. and Alvarez et al. [25, 26] on a Bioplex platform (M/S Biorad Laboratories Ltd). Protein was estimated in both plasma and adipose tissue lysate by Bicinchoninic acid method [27].

### 2.7. Plasma Lipid Profile

Levels of total cholesterol, HDL cholesterol, and triglycerides were estimated in fasting plasma using assay kits from Biosystems (Barcelona, Spain). Levels of plasma free fatty acids were determined using the enzymatic kit from Randox (Antrim, UK).

### 2.8. Fat Staining of Liver

Oil Red “O” staining of the frozen liver sections was used to demonstrate fat deposition in the liver of MnC and MnR groups of animals according to the protocol described earlier [28].

### 2.9. Fasting Plasma Glucose and Insulin

After an overnight fast, blood was withdrawn from the supraorbital sinus, and plasma glucose and insulin concentrations were determined using an enzymatic kit from Biosystems (Barcelona, Spain) and a radioimmunoassay kit from BRIT (Mumbai, India), respectively. 

### 2.10. Insulin Resistance (HOMA-IR)

Insulin resistance was assessed from fasting plasma glucose and insulin concentrations by computing the homeostasis model assessment of insulin resistance (HOMA IR) values according to the following formula:

(1) $$\begin{array}{cc} & \text{HOMA-IR}\\ & \;=\frac{\left[\text{Fasting}\;\text{insulin}\;\left(\mu \text{U}/\text{mL}\right)\times \text{Fasting}\;\text{glucose}\;\left(\text{mM}\right)\right]}{22.5}. \end{array}$$

### 2.11. Statistical Analysis

Data was analysed using SPSS version 15. Comparisons between control and MnR female WNIN rats (mothers before mating) were made by Student's *t*-test. Data collected from the offspring after weaning were analysed using one-way ANOVA followed by the post hoc multiple range test/least significance difference (LSD) test as appropriate. Wherever heterogeneity of variance was observed, differences between groups were tested by nonparametric Mann-Whitney *U* test as well as after log transformation of the data appropriately. Comparisons considered were control (MnC) versus MnR and MnR versus MnRC, MnRP, and MnRW. All values are reported as mean ± SE. Differences were considered significant only if “*P*” was <0.05.

## 3. Results

### 3.1. In the Mothers (WNIN Female Rats) 

#### 3.1.1. Growth, Mn Status, and Lipid Profile

Food intake was comparable among rats fed MnC or MnR diets, and in line with this, there was no significant difference between the two groups in their body weight gain. As expected, there was a significant decrease in blood Mn levels in MnR compared to MnC rats (Table 3). Mn restriction *per se* had no effect on plasma lipid profile compared to controls (Table 4). 

#### 3.1.2. Reproductive Performance

There was 100% conception in both MnC and MnR rats, which had comparable weight gains during pregnancy. Litter size, number of still births, mean birth weight of pups, and percentage of deaths during lactation were comparable between the two groups (Table 5).

### 3.2. In the Offspring

#### 3.2.1. Body fat%

**Figure 2:**
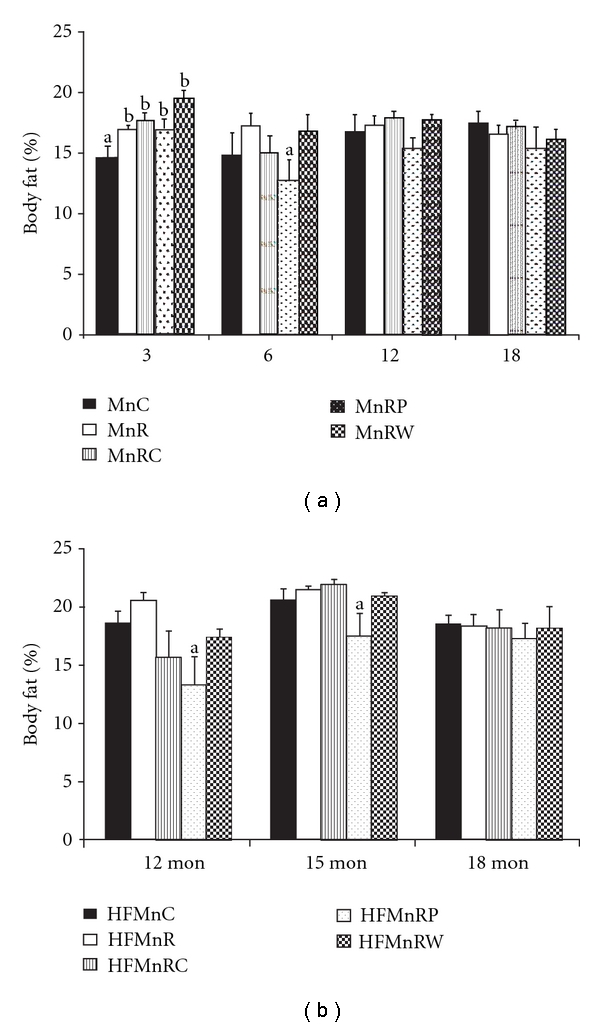
Body fat% in normal fat-fed (a) and HF-fed offspring (b) at different time points. Values are mean + SE (*n* = 6). Bars without a common letter (“a” and “b”) are significantly different at *P* < 0.05 by one-way ANOVA followed by post hoc LSD (least significant difference) test. MnC: control group, MnR: manganese restricted group, MnRC: rehabilitation from conception group, MnRP: rehabilitation from parturition group, and MnRW: rehabilitation from weaning group.

MnR offspring had significantly higher body fat% than MnC only at 3 months of age but not later (Figure 2(a)), and rehabilitation did not correct this change. Indeed there were no changes in body fat% of the HF-fed animals of all five groups at all the time points studied (Figure 2(b)).

#### 3.2.2. Adiposity Index

**Figure 3:**
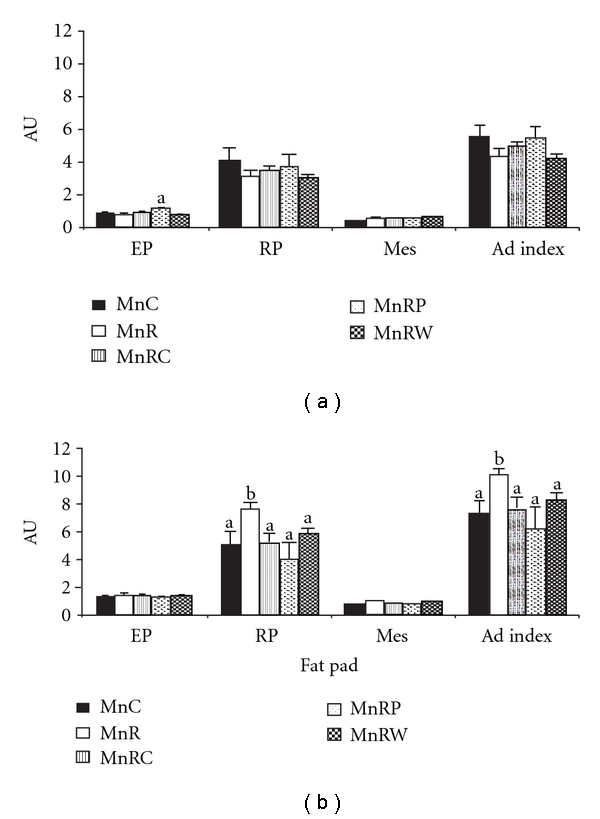
Adiposity index in normal fat-fed and HF-fed offspring at 18 mon of age. Values are mean + SE (*n* = 6).

The wet weights of the three major fat depots, namely, epididymal (EP), retroperitoneal (RP), and mesenteric (Mes) as well as the adiposity index (AI), were comparable among the five different groups of offspring fed normal fat diets (Figure 3(a)). Interestingly, the wet weight of the RP fat pad was significantly higher in HFMnR than HFMnC rats but was correctable by all the rehabilitation regimens. However, wet weight of mesenteric and epididymal fat pads were comparable between HFMnR and HFMnC offspring. In line with this, adiposity index was significantly increased in the HFMnR than HFMnC offspring (Figure 3(b)).

#### 3.2.3. Plasma Adipocytokines

TNF-*α* levels were significantly higher in MnR than MnC offspring and rehabilitation corrected the change *albeit *partially. Levels of MCP-1, Leptin, IL-6, IL-1*β* and PAI-total were comparable among the offspring of all groups. Interestingly circulating levels of MCP-1, Leptin, IL-6 and TNF-*α* were significantly higher and that of IL-1*β* significantly lower in HFMnR compared to HFMnC offspring (Table 6).

#### 3.2.4. Adipocytokines in Adipose Tissue

Adipocytokine levels in the adipose tissue lysate were in general comparable among the offspring of both the diet types (data not shown).

#### 3.2.5. Plasma Lipid Profile

**Figure 4:**
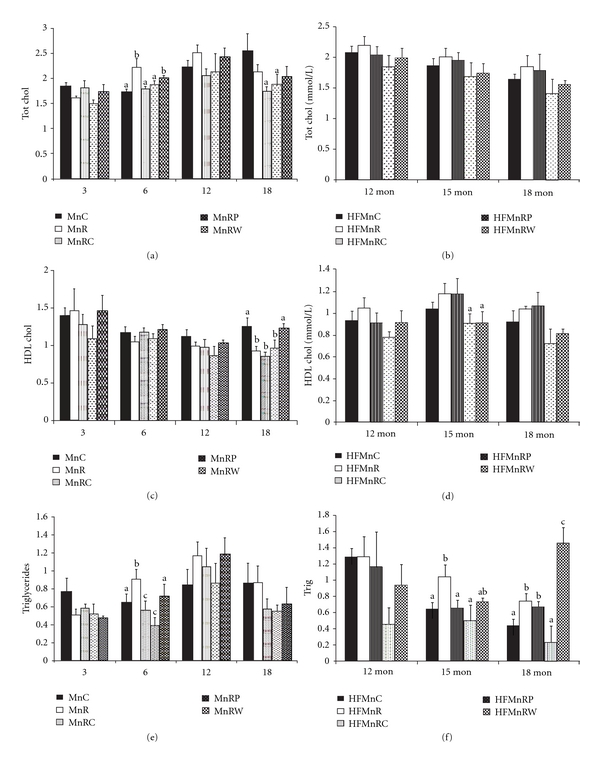
(a, b) Total cholesterol in normal fat-fed (a) and HF-fed (b) offspring at different ages. Values are mean + SE (*n* = 6). Bars without a common letter (“a” and “b”) are significantly different at *P* < 0.05 by one-way ANOVA followed by post hoc LSD (least significant difference) test. (c, d) HDL cholesterol in normal fat-fed and HF-fed offspring at different ages. Values are mean + SE (*n* = 6). Bars without a common letter (“a” and “b”) are significantly different at *P* < 0.05 by one-way ANOVA followed by post hoc LSD (least significant difference) test. (e, f) Triglyceride levels in normal fat-fed and HF-fed offspring at various time points. Values are mean + SE (*n* = 6). Bars without a common letter (“a” and “b”) are significantly different at *P* < 0.05 by one-way ANOVA followed by post hoc LSD (least significant difference) test.

Maternal Mn restriction significantly increased total cholesterol in the offspring at 6 months of age and the change was corrected by MnRC and MnRP but not MnRW (Figure 4(a)). Indeed at 18 months of age MnRC and MnRP offspring had significantly lower total cholesterol levels among all the groups. HDL cholesterol was significantly lower in MnR offspring *albeit* only at 18 months of age and surprisingly this was corrected only by MnRW (Figure 4(c)). The levels of total cholesterol and HDL cholesterol were not different among different groups of offspring fed HF diet (Figures 4(b) and 4(d)). Plasma triglycerides were significantly higher in MnR than MnC offspring only at 6 months of age but not later and here again only MnRW corrected the change (Figure 4(e)). HFMnR rats had significantly higher plasma triglyceride levels at 15 and 18 months of age (not earlier) than HFMnC which was only partially corrected by rehabilitation. Indeed at 18 months of age, HFMnRW group had significantly higher triglyceride levels than all other groups (Figure 4(f)). The levels of plasma free fatty acids were, in general, comparable among the groups of both the diet types (data not given).

#### 3.2.6. Fat Staining of Liver

**Figure 5:**
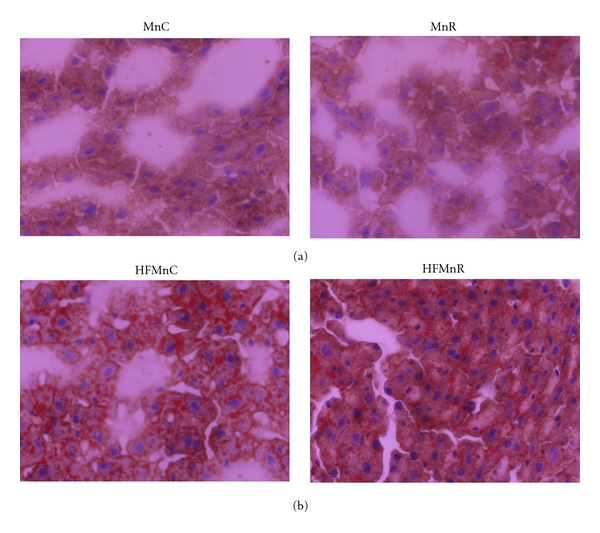
Oil red “O” staining in normal fat-fed (a) and HF-fed (b) MnC and MnR offspring at 18 months of age.

Oil red “O” staining of the frozen liver sections showed no significant difference between the MnC and MnR animals; however, the HFMnR offspring showed significantly higher fat deposition in liver compared to HFMnC (Figures 5(a) and 5(b)).

#### 3.2.7. Fasting Plasma Glucose, Insulin, and HOMA-IR

**Figure 6:**
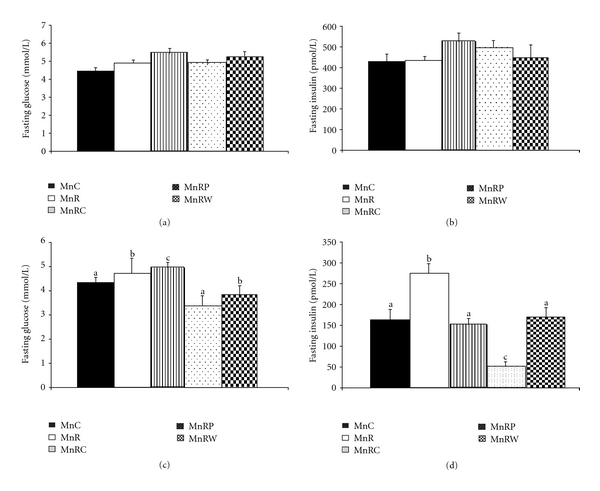
(a) Levels of fasting plasma glucose in different groups of NF-fed rat offspring at 9 months of age. (b) Levels of fasting plasma insulin in different groups of NF-fed rat offspring at 9 months of age. (c) Levels of fasting plasma glucose in different groups of HF-fed rat offspring at 18 months of age. (d) Levels of fasting plasma insulin in different groups of HF-fed rat offspring at 18 months of age.

The levels of fasting plasma glucose and insulin were comparable among the offspring of different groups fed normal fat diet at all the time points tested. A representative plot of the same at 9 months of age of the offspring is shown in Figures 6(a) and 6(b). In line with these findings, HOMA-IR values were also comparable among the groups (data not given). On the other hand, in the HF diet-fed offspring, fasting plasma glucose, insulin, and HOMA IR were significantly higher in HFMnR than HFMnC at 18 months of age, and the change was only partially corrected by rehabilitation (Figures 6(c) and 6(d)). 

## 4. Discussion

Maternal undernutrition predisposes the offspring to metabolic diseases in later life. Animal models developed so far have focused mostly on macronutrient deficiencies to understand the mechanistic basis of this relationship. In addition, the role of maternal micronutrient status in programming the foetus to adult onset diseases has not been well studied. Previous studies from our lab have shown that offspring born to micronutrient restricted rat dams were predisposed to high body adiposity and insulin resistance in their later life [4]. The present study assessed the effects of maternal, peri/postnatal Mn deficiency and effect of postnatal high-fat feeding on the development of adiposity and modulation of adipocyte metabolism in the offspring. 

Using low-trace casein (which contains negligible amounts of all trace elements) in the MnR diet, 96% deficiency of Mn was created in the restricted diet. That daily food intake was comparable between the WNIN female rats fed control and MnR diet groups is in line with the observations of Venu et al., who reported no change in food intake in Mg restricted WNIN female rats [29]. Notwithstanding, the significantly lower blood Mn levels in MnR than MnC female rats appears to be due to very low Mn content of the MnR diet and is in line with Wood who reported lower blood Mn levels in the mothers of IUGR babies [30]. Though Mn is reported to have strong causal relationship with birth, congenital, and teratogenic defects in various animal species [31, 32], that body weight gain was comparable between MnC and MnR female WNIN rats appears to be in line with studies where rat growth was not affected by Mn deficiency [33]. Although *in vitro* studies show that Mn stimulates cholesterol synthesis [34], it was surprising that cholesterol levels were comparable between the two groups of rats. Maternal Mn restriction did not influence weight gain during pregnancy, neonatal mortalities or litter size, which is in line with our observations in Mg-restricted rats reported earlier [24].

High body adiposity, particularly visceral adiposity, is the known forerunner of IR [35, 36]. However, chronic maternal Mn restriction showed only transient changes in body fat% in the NF-fed offspring and was not associated with any changes in visceral adiposity (adiposity index). It is expected that consuming high-fat diet results in obesity [37]. In line with this, it was observed in the present study that although there was no effect on body fat%* per se*, there was an increase in retroperitoneal fat pad weight as also the central/visceral adiposity in the HFMnR offspring compared to HFMnC. The increased central adiposity observed in these animals is indeed in agreement with our previous studies where maternal chromium restriction increased central adiposity in the rat offspring [38]. This observation of ours has for the first time shown the higher susceptibility of the MnR rat offspring to increased central adiposity on HF feeding in their later life as compared to their control counterparts.

Adipose tissue is an active secretor of metabolically important molecules called adipocytokines which regulate lipid/carbohydrate metabolism. In adipose tissue, TNF-*α* represses genes involved in uptake and storage of nonessential fatty acids and glucose, genes for transcription factors involved in adipogenesis and lipogenesis, and modulates the expression of several adipocytokines including adiponectin and IL-6 [39]. Leptin, which regulates the amount of body fat [40] is expressed/secreted in proportion to adipose mass, and its plasma levels are highly correlated to body fat mass [41, 42]. IL-6 expression in adipose tissue and its circulating levels are positively correlated with obesity, impaired glucose tolerance, and IR [43]. It was surprising that despite no change observed in the visceral adiposity of MnR offspring, there was a considerable increase in circulating levels of TNF-*α* in them, and this was corrected partially by rehabilitation. The proinflammatory state seen here is in line with similar changes in adipocytokine profile we reported recently in Cr-restricted rat offspring [38]. That such changes in adipocytokine profile were not seen in the adipose tissue was, however, perplexing. Taken together with the transient nature of the changes in body fat% and lack of changes in visceral adiposity, these findings appear to suggest that maternal Mn restriction may affect only the adipocyte function but not its development in the offspring. To the best of our knowledge, these are the first reports to show that maternal Mn restriction induced a proinflammatory state in the offspring although their adiposity was not affected, indicating its probable importance in regulating adipocyte function. 

Previous data has suggested that circulating leptin levels not a reflection of adiposity or energy balance, but also are strongly affected by dietary macronutrient content [44]. Most forms of rodent obesity are characterized by increased serum leptin levels and increased leptin mRNA expression in the adipose tissue [45]. The increase in leptin gene expression in rats consuming a high-fat diet has also been previously reported [46]. It was interesting that the increased visceral adiposity of HFMnR rats was associated with increased circulating levels of leptin, TNF-*α*, IL-6, and MCP-1 compared to HFMnC. Surprisingly, here again, the adipose tissue levels of adipocytokines were comparable among different groups of HF diet-fed rat offspring. Thus, this study appears to suggest that maternal Mn restriction may increase the susceptibility of the offspring to the ill effects of high-fat feeding in their later life. 

Our observation that Oil Red “O” staining of liver was comparable between the MnC and MnR groups is in line with the comparable body fat% between the two groups of rats. It was, however, interesting to note that the increased adiposity in the HFMnR rats was associated with increased lipid accumulation in liver compared to HFMnC rats, creating a “fatty liver” like condition. The increased fat deposition in the liver of HFMnR group of animals is in line with studies wherein tissues of Mn-deficient mice were shown to have enlarged deposits of abdominal fat and fatty livers [47]. Our present findings thus seem to suggest that Mn indeed has a role in determining the degree of fat deposited under high-fat fed conditions.

The transient increase in total cholesterol levels in MnR offspring (at 6 months of age) and decrease in HDL cholesterol at 18 months of age compared to controls suggest that maternal Mn restriction induces dyslipidemia and alters lipid metabolism in the offspring *albeit* transiently. It is, however, not very surprising, since studies have suggested that the HDL abnormalities in Mn-deficient rats may be in the structure of the protein component and not the cholesterol concentration *per se* [48]. It was indeed interesting to note higher plasma triglyceride levels in HFMnR compared to HFMnC rats which strongly supports the increased adiposity observed in these animals. 

 Robust evidence suggests that Mn deficiency results in a diabetes-like glucose intolerance in experimental animals. This may result from alterations in pancreatic insulin synthesis, secretion, and degradation as well as modulation of insulin action on peripheral target tissues. In contrast to these reports, we observed comparable fasting plasma glucose and insulin levels between MnC and MnR offspring fed NF diets. Interestingly, this observation is in line with our previous report that multimineral restriction *per se* in the mothers had no discernible effect on glucose tolerance or insulin resistance in the WNIN rat offspring [4] although it increased their body fat%, specially the visceral adiposity. Indeed, it is interesting that MnR offspring fed HF diet had significantly higher fasting glucose and insulin levels than corresponding controls, suggesting that maternal Mn restriction probably increased the susceptibility of the offspring to the ill effects of HF feeding specially the modulation of glucose and insulin homeostasis.

Central obesity and alteration of adipokine secretion, together with fat accumulation in different metabolically active tissues such as liver, muscle, and pancreas, constitute the pathophysiologic basis of the metabolic syndrome [49, 50]. It can thus be concluded that maternal restriction of dietary Mn, an essential trace element, is important in regulating adipocyte function in the offspring and increasing their susceptibility to increased visceral adiposity in addition to modulating their glucose and insulin homeostasis specially when superimposed with a high-fat diet in their later life. 

## 5. Conclusion

This study has, for the first time, demonstrated that maternal Mn restriction predisposes the offspring to increased central adiposity, fat deposition in liver, induction of a proinflammatory state, altered adipocyte function, dyslipidemia, and altered homeostasis of glucose and insulin possibly leading to a metabolic syndrome-like situation specially when challenged with high-fat diet in later life, a situation that prevails currently in a developing country like India.

## Figures and Tables

**Table 1:** Comparison of the composition of the AIN-93G diet and the high-fat diet.

Diet ingredients	G/Kg diet in AIN 93G diet	G/Kg diet in High-fat diet
Starch	529.5 g	425 g
Sucrose	100 g	100 g
Cellulose	50 g	50 g
Casein (20%)	200 g	200 g
Groundnut oil	70 g	175 g
Mineral mix	35 g	35 g
Vitamin mix	10 g	10 g
L-cystine	3 g	3 g
Choline	2.5 g	2.5 g

**Table 2:** Comparison of energies obtained from different diets.

Diet component	Energy obtained from AIN 93G diet	Energy obtained from high-fat diet
Carbohydrates	64.8%	50.0%
Proteins	19.24%	19.24%
Fat	16%	32.72%

**Table 3:** Food intake and body weight gain of WNIN female rats fed control and Mn-restricted diets for 16 weeks before mating.

Parameters	MnC	MnR
Food intake (g)	10.5 ± 0.323	10.1 ± 0.173
Body weight gain (g)	107 ± 1.79	107 ± 0.76
Blood Mn conc (*μ*g/L)	14.2 ± 4.02	9.01 ± 2.31*

Values are means ± SE, *n* = 6; *P < 0.05 using Student's *t*-test.

**Table 4:** Plasma lipid profile of WNIN female rats fed control and Mn-restricted diets for 16 weeks before mating.

Parameters	MnC	MnR
Cholesterol (mmol/L)	1.60 ± 0.058	1.51 ± 0.086
HDL cholesterol (mmol/L)	1.15 ± 0.076	1.02 ± 0.054
Triglycerides (mmol/L)	0.653 ± 0.057	0.543 ± 0.028

Values are means ± SE (*n* = 6).

**Table 5:** Reproductive performance of the female WNIN rats fed different diets.

Parameter	MnC	MnR	MnRC
Body wt before mating	182 ± 6.52	180 ± 2.39	198 ± 6.05
% conceived	100	100	100
Wt gain during pregnancy (g)	90.9 ± 6.20	96.1 ± 1.85	92.5 ± 6.75
% aborted	Nil	Nil	Nil
Litter size	3–11	6–13	5–11
Still births	Nil	Nil	Nil
Deaths during lactation	Nil	Nil	Nil
Mean birth wt (g)	5.60 ± 0.204	5.56 ± 0.086	5.63 ± 0.120

Values are mean ± SE, *n* = 6.

**Table 6:** Plasma adipocytokines status in offspring at 18 months of age.

		*Normal-fat-fed offspring*			
	MnC	MnR	MnRC	MnRP	MnRW
MCP-1 (pg/mL)	174 ± 51.4	197 ± 88.9	811 ± 555	78.9 ± 4.69	255 ± 131
Leptin (pg/mL)	2727 ± 705	2226 ± 391	2553 ± 708	3279 ± 1445	2399 ± 533
IL-1*β* (pg/mL)	31.5 ± 9.14	78.9 ± 27.9	84.3 ± 48.2	27.3 ± 10.5	30.6 ± 9.15
IL-6 (pg/mL)	12.9 ± 0.036	35.3 ± 11.2	68.6 ± 32.5	33.6 ± 0.69	24.5 ± 6.47
TNF-*α* (pg/mL)	1.62 ± 0.380^a^	16.3 ± 0.880^b^	13.0 ± 0.023^b^	2.33 ± 0.330^a^	12.3 ± 0.330^b^

*High-fat-fed offspring*					
	MnC	MnR	MnRC	MnRP	MnRW

MCP-1 (pg/mL)	36.3 ± 0.850^a^	54.1 ± 2.07^b^	98.8 ± 1.34^c^	51.0 ± 0.640^b^	119 ± 30.9^c^
Leptin (pg/mL)	2344 ± 315^a^	4742 ± 164^b^	3315 ± 603^a^	1898 ± 956^a^	2376 ± 1028^a^
IL-1*β* (pg/mL)	44.9 ± 9.54^a^	13.3 ± 1.96^b^	24.8 ± 2.78^b,c^	23.3 ± 6.75^b^	94.5 ± 3.22^c^
IL-6 (pg/mL)	20.9 ± 0.580^a^	74.4 ± 1.80^b^	10.7 ± 0.650^c^	44.6 ± 5.88^c^	49.9 ± 0.570^c^
TNF-*α* (pg/mL)	1.33 ± 0.330^a^	4.67 ± 0.880^b^	2.33 ± 0.330^a,b^	2.00 ± 0.580^a^	1.67 ± 0.670^a^

Values are mean ± SE (*n* = 6). Values bearing different superscript in a given row are significantly different from others by one-way ANOVA/multiple range test/least significant difference test. MnC: control group, MnR: manganese restricted group, MnRC: rehabilitation from conception group, MnRP: rehabilitation from parturition group, and MnRW: rehabilitation from weaning group. a,b and c: means without a common superscript are significantly different by One way ANOVA. b: *P* < 0.01 and c: *P* < 0.001.
